# Prison Mental Health and Substance Misuse in Wales–Informing a Network

**DOI:** 10.1192/bjo.2026.11528

**Published:** 2026-06-30

**Authors:** Alka Ahuja, Ollie John, Joanna Dainton, Adrian Clarke

**Affiliations:** 1 Royal College of Psychiatrists Wales, Cardiff, United Kingdom; 2 NHS Wales, Cardiff, United Kingdom

## Abstract

**Aims:**

The primary objective of this baseline audit was to evaluate the performance of mentalhealth and substance misuse services across all five Welsh prison sites against evidence-based quality standards.

The study sought to identify models of care, highlight areas of best practice, pinpoint thematic gaps requiring additional strategic resource or funding, and inform the development of a future network for prison mental health and substance misuse services in Wales.

**Methods:**

The audit utilised a peer-review methodology developed by the Quality Network for Prison Mental Health Services (QNPMHS). Each service underwent a two-month self-assessment against 238 standards, followed by an intensive in-person review visit between September 2024 and July 2025.

Data collection involved 92 semi-structured interviews with frontline staff, prison colleagues, and patients, alongside environmental tours and documentation reviews. Compliance was measured against Mental Health (MH) standards and the 2024 Substance Misuse Treatment Framework.

Visits were undertaken by the College Centre for Quality Improvement (CCQI), commissioned by RCPsych Wales as part of the Prison partnership agreement between Welsh Government and HMPPS.

**Results:**

The audit revealed variable compliance levels, with average scores of 62.8% for mental health and 57.7% for substance misuse standards. Key findings included:

- Service Models: Most services follow a nurse-led model, with significant gaps in access to psychology, occupational therapy, and psychiatry.
- Substance Misuse: Only one prison (HMP Berwyn) maintained a full clinical SM team, while others relied on single practitioners managing high caseloads.
- Neurodiversity: While “whole-prison” neurodiversity support managers exist, healthcare teams lack the commissioning to provide formal assessments or diagnoses for ADHD and Autism.
- Operational Barriers: Challenges included a lack of confidential consultation rooms, insufficient IT resources, and inconsistent provision of monthly clinical supervision.

**Conclusion:**

While pockets of good practice exist, particularly in collaborative duty worker roles, the Welsh prison estate faces systemic challenges regarding clinical multidisciplinary input and specialist pathways.

The report recommends immediate investment in specialist Substance Misuse staff, formalising neurodivergent care pathways, and enhancing clinical working environments.

Implementing adapted assessments for older adults and ensuring monthly clinical supervision are critical priorities to improve the quality and safety of care.

The review provides critical insight, informing the development of a future network for prison mental health and substance misuse services in Wales, commissioned by Welsh Government.

